# Draft Genome Sequence of *Corynebacterium pseudotuberculosis* Strain PA07 Biovar *ovis*, Isolated from a Sheep Udder in Amazonia

**DOI:** 10.1128/genomeA.00040-17

**Published:** 2017-03-23

**Authors:** Fabrício Almeida Araújo, Joana Montezano Marques, Vitória Almeida Gonçalves de Moura, Maria Paula Cruz Schneider, Soraya Silva Andrade, Alyne Cristina Sodré Lima, Luis Carlos Guimarães, Adriana Ribeiro Carneiro Folador, Artur Silva, Rommel T. J. Ramos

**Affiliations:** University of Pará, Center of Genomics and System Biology, Laboratory of Genomic and Bioinformatics, Belém, Pará, Brazil

## Abstract

In this work, we present the draft genome sequence of *Corynebacterium pseudotuberculosis* strain PA07 biovar *ovis*, isolated from a caseous secretion from a sheep udder in Pará, Brazil. The genome contains 2,320,235 bp, 52.2% G+C content, 2,191 coding sequences (CDSs), five pseudogenes, 48 tRNAs, and three rRNAs.

## GENOME ANNOUNCEMENT

*Corynebacterium pseudotuberculosis* is a Gram-positive, facultative intracellular, pleomorphic, nonsporulating, noncapsulated, nonmotile bacterium that, together with *Mycobacterium*, *Nocardia*, and *Rhodococcus*, forms the CMNR group which shares specifics characteristics, such as (i) high G+C content and (ii) specific cell wall organization, characterized by the presence of peptidoglycan, arabinogalactan, and mycolic acids (1, 2).

*C. pseudotuberculosis* is the etiological agent of caseous lymphadenitis (CLA), ulcerative lymphangitis, mastitis (clinical and subclinical), and ulcerative dermatitis. CLA is characterized by the formation of abscesses in the superficial lymph nodes and subcutaneous tissues (3, 4). CLA is a widespread disease that has been reported all over the world (5). This disease is associated with direct economic losses due to the progressive reduction in weight gain, depreciated wool and skin, reduced milk production, and eventually death caused by toxemia of the infected animals. Moreover, this bacterium presents significant zoonotic potential (5).

*C. pseudotuberculosis* presents some variable tools for persistence and escape from the immune system of the host (3). Thus, the molecular study of the genome of this pathogen and the search for new target genes is an important step for vaccine development (6, 7).

Herein, we present the draft genome of *C. pseudotuberculosis* strain PA07 biovar *ovis*, isolated from a Santa Inês sheep udder in Pará, Brazil. To the best of our knowledge, we report the first recovery of *C. pseudotuberculosis* from an abscess located in sheep udder in the state of Pará. This strain is part of the collection of Laboratory of Genomics and System Biology located at the Federal University of Pará, Belém, Pará, Brazil.

The genome sequencing was performed using an Ion Torrent personal genome machine (PGM) (Thermo, Fisher) with a fragment library. The *de novo* assembly strategy was performed using SPAdes genome assembler software version 3.9.0 (8). The assembly generated seven contigs with 2,320,235 bp. The contigs were automatically annotated using Rapid Annotations using Subsystems Technology (RAST) (9). The genome has 2,191 coding sequences (CDSs), five pseudogenes, 48 tRNAs, three rRNAs, and a G+C content of 52.2%.

### Accession number(s).

The *C. pseudotuberculosis* whole-genome shotgun project has been deposited at DDBJ/ENA/GenBank under the accession no. MQVG00000000. The version described in this paper is MQVG01000000.

## References

[1] Rebouças MF, Loureiro D, Bastos BL, Moura-Costa LF, Hanna SA, Azevedo V, Meyer R, Portela RW 2013 Development of an indirect ELISA to detect *Corynebacterium pseudotuberculosis* specific antibodies in sheep employing T1 strain culture supernatant as antigen. Pesq Vet Bras 33:1296–1302. doi:10.1590/S0100-736X2013001100002.

[2] Dorella FA, Pacheco LGC, Oliveira SC, Miyoshi A, Azevedo V 2006 *Corynebacterium pseudotuberculosis*: microbiology, biochemical properties, pathogenesis and molecular studies of virulence. Vet Res 37:201–218. doi:10.1051/vetres:2005056. 16472520

[3] Baird GJ, Fontaine MC 2007 *Corynebacterium pseudotuberculosis* and its role in ovine caseous lymphadenitis. J Comp Pathol 137:179–210. doi:10.1016/j.jcpa.2007.07.002. 17826790

[4] Yeruham I, Elad D, Friedman S, Perl S 2003 *Corynebacterium pseudotuberculosis* infection in Israeli dairy cattle. Epidemiol Infect 131:947–955. doi:10.1017/S095026880300894X. 14596537 PMC2870040

[5] Soares SC, Silva A, Trost E, Blom J, Ramos R, Carneiro A, Ali A, Santos AR, Pinto AC, Diniz C, Barbosa EG, Dorella FA, Aburjaile F, Rocha FS, Nascimento KK, Guimarães LC, Almeida S, Hassan SS, Bakhtiar SM, Pereira UP, Abreu VAC, Schneider MPC, Miyoshi A, Tauch A, Azevedo V 2013 The pan-genome of the animal pathogen *Corynebacterium pseudotuberculosis* reveals differences in genome plasticity between the biovar *ovis* and *equi* strains. PLoS One 8:e53818. doi:10.1371/journal.pone.0053818. 23342011 PMC3544762

[6] D’Afonseca V, Moraes PM, Dorella FA, Pacheco LG, Meyer R, Portela RW, Miyoshi A, Azevedo V 2008 A description of genes of *Corynebacterium pseudotuberculosis* useful in diagnostics and vaccine applications. Genet Mol Res 7:252–260. doi:10.4238/vol7-1gmr438. 18551390

[7] Dorella FA, Gala-Garcia A, Pinto AC, Sarrouh B, Antunes CA, Ribeiro D, Aburjaile FF, Fiaux KK, Guimarães LC, Seyffert N, El-Aouar RA, Silva R, Hassan SS, Castro TLP, Marques WS, Ramos R, Carneiro A, de Sá P, Miyoshi A, Azevedo V, Silva A 2013 Progression of “OMICS” methodologies for understanding the pathogenicity of *Corynebacterium pseudotuberculosis*: the Brazilian experience. Comput Struct Biotechnol J 6:1–7. doi:10.5936/csbj.201303013. PMC3962224 24688721

[8] Bankevich A, Nurk S, Antipov D, Gurevich AA, Dvorkin M, Kulikov AS, Lesin VM, Nikolenko SI, Pham S, Prjibelski AD, Pyshkin AV, Sirotkin AV, Vyahhi N, Tesler G, Alekseyev MA, Pevzner PA 2012 SPAdes: a new genome assembly algorithm and its applications to single- cell sequencing. J Comput Biol 19:455–477. doi:10.1089/cmb.2012.0021. 22506599 PMC3342519

[9] Aziz RK, Bartels D, Best AA, DeJongh M, Disz T, Edwards RA, Formsma K, Gerdes S, Glass EM, Kubal M, Meyer F, Olsen GJ, Olson R, Osterman AL, Overbeek RA, McNeil LK, Paarmann D, Paczian T, Parrello B, Pusch GD, Reich C, Stevens R, Vassieva O, Vonstein V, Wilke A, Zagnitko O 2008 The RAST server: Rapid Annotations using Subsystems Technology. BMC Genomics 9:75. doi:10.1186/1471-2164-9-75. 18261238 PMC2265698

